# Bending the arc of COVID-19 through a principled food systems approach

**DOI:** 10.1007/s10460-020-10048-2

**Published:** 2020-05-31

**Authors:** Ruth Richardson

**Affiliations:** Global Alliance for the Future of Food, Toronto, Canada

According to reports, the first case of COVID-19 can be traced back to 17 November in China, not more than 5 months ago. As the pandemic quickly spreads, with what is now estimated at half of the world's population in lockdown, there has been much discussion on both cause and effect of this coronavirus.

On the *cause* of COVID-19, the evidence predominantly points to food systems. First, deforestation due to commercial agriculture enables the spread of coronaviruses, or zoonotic diseases, by increasing the likelihood of human-wildlife interaction. Second, large-scale industrial livestock production creates the conditions for the propagation of zoonotic viruses due to the confinement of large numbers of animals in small spaces, narrowed genetic diversity, fast animal turnover, and habitat fragmentation through expansion of livestock production (IPES-Food 2020).

On the question of the *effects* that are being, and will be, witnessed, one just needs to look at the headlines to catch a glimpse of the short- and long-term impacts that reverberate in COVID-19’s wake: death, unemployment, market instability, mental health, substance abuse, and more. The impacts are no less significant as they relate to food systems: supply chain interruptions, food insecurity, the wasting of vast quantities of commodities such as milk and eggs, the increase in consumption of highly-processed foods, the collapse of markets impacting the livelihoods of producers around the world. What’s more, women, smallholder farmers, vulnerable populations, and poor and marginalized communities are at the sharp end of these impacts and carry an unequal burden of COVID-19’s devasting effects.

Both cause and effect point to an urgent need to radically transform our food systems. We must re-imagine and re-build more equitable, secure, and sustainable food systems so that we: minimize the potential negative impacts of the ways we produce, process, and consume our food from zoonotic disease to greenhouse gas emissions to rates of non-communicable diseases; and, equally, strengthen our food systems’ ability to withstand—remain resilient to—other shocks such as pandemics, extreme weather events, climate change induced temperature increases, migration, and sea-level rise.

Transforming a system—especially one resistant to change—is daunting. Systems change theory argues that a high leverage point is changing the *goals* of the system. Whether you call them goals, values, or principles they define what the system aims to achieve. By changing the goals of the system, you change the system.

In the face of COVID-19 what food systems transformation requires is that we uphold the goals of diversity, resilience, renewability, equity, inclusion, health, and interconnectedness—the set of goals that the Global Alliance for the Future of Food developed in 2013 to define its vision for food systems transformation. When taken together, these goals, or principles, help us to see food systems in necessary and powerful new ways and to make choices about how to realize a more positive future of food. They provide guidance, inform decisions, and tell us how to act into a new, better future.

Amidst the pandemic, there is an initiative underway in the state of Andhra Pradesh in south-east India called Zero Budget Natural Farming (ZBNF). ZNBF has turned its back on the old goals of an out-dated agricultural vision singularly focused on efficiency, yield, and income. Instead, ZBNF promotes a beautifully complete vision of the future of food on this planet based on a set of mutually reinforcing, equally important principles including diversity, resilience, equity, and inclusion. Its practices are already proving effective in withstanding cyclones, conserving water, diversifying nutritional staples, improving household incomes, and fostering new networks of social support.

Chris Kutarna, Oxford Martin School Fellow and author of *Age of Discovery: Navigating the Storms of Our Second Renaissance,* argues that principles, such as those espoused by ZBNF, are not just pretty words, “they are social technologies that help us to thrive through periods of disruption, of upheaval, of rapid change. It sounds simple but it isn’t easy. But if we can practice these virtues, strengthen these virtues … then that’s how we bend the arc of all these events in a positive direction.”

Now is the time, more than ever, to embrace these values, practice these values, fight for these values. Food systems transformation is one of the most defining issues of our time. For this transformation to happen with the magnitude and speed required means we have to get the goals of the system right. Only then can we can bring to life food systems that feed everyone well, that honour our planet and the other species that share it with us, that mitigate the frightening implications of global pandemics—and that leave no one behind.

